# Inulin reduces visceral adipose tissue mass and improves glucose tolerance through altering gut metabolites

**DOI:** 10.1186/s12986-022-00685-1

**Published:** 2022-07-28

**Authors:** Hanako Nakajima, Naoko Nakanishi, Tomoki Miyoshi, Takuro Okamura, Yoshitaka Hashimoto, Takafumi Senmaru, Saori Majima, Emi Ushigome, Mai Asano, Mihoko Yamaguchi, Jun Mori, Norihiro Sakui, Ryoichi Sasano, Masahiro Yamazaki, Masahide Hamaguchi, Michiaki Fukui

**Affiliations:** 1 Department of Endocrinology and Metabolism, Graduate School of Medical Science, Kyoto Prefectural University of Medicine, 465 Kajii-cho, Kawaramachi-Hirokoji, Kamigyo-ku, Kyoto, 602-8566 Japan; 2 Department of Pediatrics, Graduate School of Medical Science, Kyoto Prefectural University of Medicine, Kyoto, Japan; 3 AiSTI SCIENCE Co., Ltd, Wakayama, Japan; 4 Agilent Technologies Japan, Ltd, Tokyo, Japan

**Keywords:** Inulin, Metabolomics analysis, Short-chain fatty acids, Succinic acid, Visceral adipose tissue

## Abstract

**Aim:**

Inulin, a soluble dietary fiber, is a source of energy for the host while the metabolites, such as short-chain fatty acids (SCFAs), produced in the gut through bacterial fermentation exerts the anti-obesity effect. In this study, we aimed to apply a metabolomics approach and clarify the role of this soluble dietary fiber on glucose and lipid metabolism under the calorie-matched condition.

**Materials and methods:**

Eight-week-old male C57BL/6J mice were fed a high-fat/high-sucrose based diet containing maltodextrin or inulin for 12 weeks through calorie-matched pair feeding. We evaluated glucose tolerance, and energy expenditure using indirect calorimetry, comprehensive metabolites in the content of jejunum, feces, and portal vein serum using gas chromatography-mass spectrometry, and histological changes in the adipose tissue.

**Results:**

The inulin group exhibited reduced visceral adipose tissue and smaller size of visceral adipocyte. It also exhibited improved glucose tolerance and an increase in energy expenditure. Reflecting the results of fermentation, the metabolomics analysis revealed an increase in the succinic acid and SCFA contents in both feces and portal vein serum in the inulin group.

**Conclusions:**

Inulin altered the gut metabolites and reduced visceral adipose tissue, thereby resulting in improved glucose tolerance.

**Supplementary Information:**

The online version contains supplementary material available at 10.1186/s12986-022-00685-1.

## Introduction

In recent years, it has been demonstrated that the intestinal microbiota and its metabolites are involved in lifestyle-related diseases, including type 2 diabetes and obesity, and the importance of dietary habits that affect these diseases has gained attention [1]. Various metabolites, including short-chain fatty acids (SCFAs), are produced from the dietary fiber through fermentation mediated by the intestinal microbiota. SCFAs produced upon intake of soluble dietary fiber play a crucial role as an energy resource for the host and are known to form the basis for lipid synthesis [2, 3] in the peripheral tissue. Conversely, a host energy control mechanism mediated via G protein-coupled receptors (GPCRs) has been reported, and its suppressive effect on adipose fat accumulation has received attention [4–6].

Dietary fiber fermentation not only produces SCFAs but also succinic acid, an organic acid, as a metabolite [7]. Succinic acid has been reported to activate thermogenesis and increases energy expenditure in adipose tissue and exert an effect on whole-body metabolism against diet-induced obesity mouse, such as the suppression of weight gain and improvement of glucose tolerance [8].

Inulin, a soluble dietary fiber, which has an energy content of 1.5 kcal/g [9] is known to promote SCFAs production starting from the proximal to distal colon [10]. Moreover, it has been shown that inulin supplementation exerts a prebiotic anti-obesity, such as the improvement of obesity, blood glucose level, and lipid metabolism. Previous reports have revealed the effect of inulin supplementation on glucose and lipid metabolism in genetically obese mice [11], or wild-type mice [12, 13] or rats [14] after induction of dietary obesity. Human studies [15, 16] have also shown that inulin intake improves glycolipid metabolism, as it is fermented into SCFAs by gut microbiota in the situation of energy excess due to the high-fat and high-sucrose diet (HFHSD), or in the presence of glucose intolerance or obesity.

Therefore, inulin is an interesting compound owing to its dual function. It is an energy source for the host while it exerts an anti-obesity effect. To deeply investigate its duality, considering the calorie of inulin, we conducted a calorie-matched pair feeding experiment under a high-fat, and high-sucrose diet. Also, comprehensive metabolomics analysis was performed to assess the changes in the metabolites in a systemic manner to clarify the underlying mechanism through which the soluble dietary fiber improves the glucose and lipid metabolism.

## Materials and methods

### Animals and diet composition

All experimental procedures were approved by the Committee for Animal Research, Kyoto Prefectural University of Medicine (Permit number: 2020-50). Seven-week-old male C57BL/6J mice were purchased from Shimizu Laboratory Supplies (Kyoto, Japan) and acclimated under a controlled environment (12 h light/12 h dark cycle; temperature, 22–24 °C; and humidity, 30–60%) with free access to water in the animal facility of the Kyoto Prefectural University of Medicine. The mice were randomly divided into two groups at the age of 8 weeks and received either high-fat and high-sucrose based control diet (Control) or high-fat and high-sucrose based inulin diet (Inulin) for 12 weeks (n = 12 per group). The diets were formulated based on the high-fat/high-sucrose diet (D12327, Research Diets Inc., New Brunswick, NJ, USA) and modified to contain equivalent compositions of fat and sucrose in both Control and Inulin diets. The composition of experimental diets was 40% kcal fat, 32% kcal sucrose, 6% cellulose, and 7.5% kcal maltodextrin for the Control diet (D19100801, Research Diets, New Brunswick, NJ), and 40% kcal fat, 32% kcal sucrose, 6% cellulose, and 7.5% kcal inulin for the Inulin diet (D19100802, Research Diets, New Brunswick, NJ). Further details of both the diets are presented in Additional file 1: Table S1. The body weight was examined weekly for 12 weeks, and food intake was measured every day. The mice were carefully paired-fed to equalize the intake of total calories.

The mice at the age of 20 weeks were sacrificed after fasting for 3 h (n = 6 per group) or in the postprandial state (n = 6 per group) through administering 0.3 mg/kg of medetomidine, 4.0 mg/kg of midazolam, and 5.0 mg/kg of butorphanol [17]. The feces samples were collected from individual mice through placing them into empty cages. The blood samples were collected from the portal vein in the postprandial state or cardiac cavity after fasting for 3 h, and then the plasma was separated through centrifugation (5000 rpm, 20 min, 4 °C). Epididymal and subcutaneous white adipose tissues were harvested and weighed. The content in the jejunum was collected. Adipose tissues, feces, and plasma samples were stored at − 80 °C until further use.

### Indirect calorimetry

In vivo indirect open-circuit calorimetry analysis was performed when the mice were at the age of 18 weeks. The rates of oxygen consumption (VO_2_) and carbon dioxide production (VCO_2_) were assessed for 48 h during 12-h light/12-h dark cycles at the inlets and outlets of the sealed chambers with the O_2_/CO_2_ metabolism measurement system for the small animals (MM202R; Muromachi Kikai Co., Ltd., Tokyo, Japan). A constant air flow (0.6 L/min) was drawn through the chamber to maintain CO_2_ concentration below 0.5%. The system was controlled under a strict 12-h light/12-h dark cycle in atmospheric conditions of 22 °C and 30–60% humidity. Throughout the experiments, the mice had ad libitum access to food and water. Respiratory quotients (RQ) and energy expenditure [18] were calculated based on VO_2_ and VCO_2_ values.

### Intraperitoneal glucose tolerance test (IPGTT)

In 20-week-old mice, the IPGTT was performed after fasting for 16 h. Blood glucose levels were assessed in the drops of blood collected at the following time points: 0, 30, 60, and 120 min after the intraperitoneal injection of glucose (2 g/kg body weight). The glucose concentration was measured using a glucometer (Glutest Neo Alpha; Sanwa Kagaku Kenkyusho, Nagoya, Japan). The area under the curve (AUC) of the IPGTT results was analyzed.

### Histological examination

The samples of epididymal white adipose tissue (eWAT) as visceral adipose tissue and subcutaneous white adipose tissue (sWAT) were fixed with 4% paraformaldehyde, and embedded in paraffin. They were sectioned into 7-mm thick slices that were stained with hematoxylin and eosin for microscopic examination. The average cell area and lipid droplets per cell were measured using the ImageJ software according to the method described in the previous study [19]. After deparaffinization using xylene and ethanol, immunolocalization was performed in the paraffin sections of eWAT and sWAT. Primary antibodies (4 °C, 12 h) and secondary antibodies (room temperature, 1 h) were applied to the slides upon dilution with PBS. Primary antibody used was Rabbit anti-UCP1 (Abcam, 1:250), and secondary antibodies used were Alexa 488-conjugated (Invitrogen) and HRP conjugated (DAKO). Biotinylated IB4 (Vector Labs, 1:50) was used with Alexa 488-conjugated streptavidin. Nuclei were stained with 4′,6-diamidino-2-phenylindole (DAPI) (Roche). Images were acquired using a BZ-X710 fluorescence microscope (Keyence, Osaka, Japan). For quantification, at least ten 10 × view fields per sample were analyzed. UCP1 expression in the white adipose tissues were quantified through measuring the cumulative pixel intensity in multiple fields of view using the ImageJ software.

### Measurement of metabolites in the content of jejunum, feces, and portal vein serum

Amino acids, organic acids, and SCFAs in the content of jejunum, feces, and portal vein serum were analyzed using gas chromatography-mass spectrometry (GC/MS) on an Agilent 7890B/7000D System (Agilent Technologies, Santa Clara, CA, USA). The feces (20 mg) and serum (50 µL) samples were added in 500 μL of acetonitrile and 500 μL of diluted water and grinded in a ball mill at 4000 rpm for 2 min. Then, the samples were shaken at 1000 rpm for 30 min at 37 °C, and centrifuged at 14,000 rpm for 3 min at room temperature. The supernatant (500 uL) was separated and added in 500 μL of acetonitrile, and further shaken at 1000 rpm for 3 min at 37 °C. Then, the samples were centrifuged at room temperature for 3 min at 14,000 rpm, and adjusted for pH to 8 using 0.1 mol/L NaOH.

The concentrations of amino acids, organic acids, and SCFAs were then determined through GC/MS using the following on-line solid phase extraction (SPE) method. In the SPE-GC system SGI-M100 (AiSTI SCIENCE, Wakayama, Japan), SPE and injection into the GC/MS system are automatically performed after the sample has been added to the vial and set on the autosampler tray. Flash-SPE ACXs (AiSTI SCIENCE) were used for the solid stratification. For measuring the level of amino acids and organic acids, 50 µL aliquots of each of the aforementioned sample extracts were loaded onto the solid phase and washed with acetonitrile and water (1:1). Then, the samples were dehydrated with acetonitrile and impregnated with 4 μL of 0.5% methoxyamine-pyridine solution. Then, N-methyl-N-trimethylsilyltrifluoroacetamide (MSTFA) was supplied to the solid phase to perform methoxylation and trimethylsilylation while derivatization, and eluted with hexane. The final product was injected through PTV injector, LVI-S250 (AiSTI SCIENCE), and the temperature was maintained at 220 °C for 0.5 min, increased gradually 50 °C/min to 290 °C, and then held there for 16 min. The samples were loaded onto a capillary column, Vf-5 ms (30 m × 0.25 mm [inner diameter] × 0.25 μm [membrane thickness]; Agilent Technologies). The column temperature was maintained at 80 °C for 3 min, then increased gradually by 25 °C/min to 190 °C, by 3 °C/min to 220 °C and by 15 °C/min to 310 °C, which was held there for 4.6 min. The sample was injected in the split mode at a split ratio of 50:1. As for measuring SCFA, 50 μL aliquots of each of the aforementioned sample extracts were loaded onto the solid phase and washed with acetonitrile and water (1:1). Then, the samples were dehydrated with acetone and impregnated with 4 μL of *N*-*tert*-butyldimethylsilyl-*N*- methyltrifluoroacetamide (MTBSTFA)-toluene solution (1:3) and eluted with hexane after derivatization on the solid phase. The final product was injected through the PTV injector, LVI-S250, and the temperature was maintained at 150 °C for 0.5 min, increased gradually 25 °C/min to 290 °C, and then held there for 16 min. The samples were loaded onto a capillary column, Vf-5 ms (30 m × 0.25 mm [inner diameter] × 0.25 μm [membrane thickness]; Agilent Technologies). The column temperature was maintained at 60 °C for 3 min, increased gradually by 10 °C/min to 100 °C and 20 °C/min to 310 °C, and then held there for 7 min. The sample was injected in the split mode at a split ratio of 20:1. Each amino acid, organic acid, and SCFA were detected in the scan mode (m/z; 70–470). All results were normalized to the peak height of norleucine, adipic acid, and tetradeuteroacetic acid of 0.01 mM for each amino acid, organic acid and SCFA, respectively.

### Measurement of lipidome in eWAT

The composition of fatty acids in murine adipose tissue was assessed using GC/MS, Agilent 7890B/7000D (Agilent Technologies, Santa Clara, CA). Briefly, the adipose tissue sample (15 mg) was methylated using the fatty acid methylation kit (Nacalai Tesque, Kyoto, Japan). The samples were loaded onto a capillary column, Vf-5 ms (30 m × 0.25 mm [inner diameter] × 0.25 μm [membrane thickness]; Agilent Technologies). The column temperature was maintained at 80 °C for 3 min, then increased gradually by 25 °C/min to 190 °C, by 3 °C/min to 220 °C and by 15 °C/min to 310 °C, which was held there for 4.6 min. The sample was injected in the split mode with a split ratio of 5:1. Each fatty acid methyl ester was detected in the selected ion monitoring mode. All results were normalized to the peak height of the C17:0 internal standard.

### Statistical analysis

The data were analyzed using JMP version 13.0 software (SAS, Cary, NC). Statistical significance of differences between groups were determined using unpaired Student’s t test and two-way repeated-measures analysis of variance was performed to evaluate the body weight change. Analysis of covariance (ANCOVA) was used to evaluate energy expenditure [20]. A *p* < 0.05 were considered statistically significant. Figures were generated using the GraphPad Prism software Version 8.0 (San Diego, CA, USA).

## Results

### Inulin reduced increase in visceral adipose tissue

**Fig. 1:**
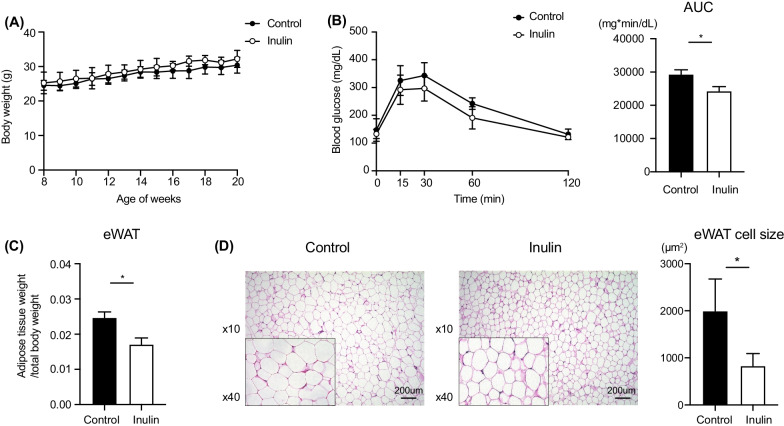
Effects of inulin on body weight gain, adipose tissue weight, and blood glucose in mice. **A** Body weight gain. Control: n = 12; Inulin group: n = 12. **B** Glucose tolerance test. The area under the curve (AUC) over the course of 120 min in each experiment was averaged. **C** Ratio of the weight of epididymal (eWAT) per body weight. **D** Histological examination of epididymal white adipose tissues (eWAT) using hematoxylin and eosin (H&E) staining. Scale bar = 200 µm. Area of average white adipocyte size in eWAT. Control: n = 6; Inulin group: n = 6. Data are expressed as means ± SEM. *p < 0.05

To investigate the effect of inulin treatment on metabolic disorders, we examined the body weight, daily food intake, and glycolipid metabolism related parameters. Initial body weight was comparable between the two groups. After being fed with the control and inulin containing HFHSD for 12 weeks, the body weight change of the mice remained unaffected (Fig. 1A). Two-way repeated-measures analysis of variance revealed the body weight change between age of 8 weeks and 12 weeks were not different between the two groups (*p* = 0.314). The two groups were strictly pair-fed, and the caloric intake of both the groups was the same as shown in Additional file 2: Figure S1A. The AUC of IPGTT in the inulin group were significantly smaller compared with that in the control group (Fig. 1B).

Figure 1C indicates the ratio of eWAT to body weight was significantly lower in the inulin group than that in the control group (*p* = 0.015). Moreover, the size of adipocyte in eWAT was significantly smaller in the inulin group compared to the control group (Fig. 1D), however, there was no change in sWAT (Additional file 2: Figure S1B).

### Inulin activates energy metabolism

**Fig. 2:**
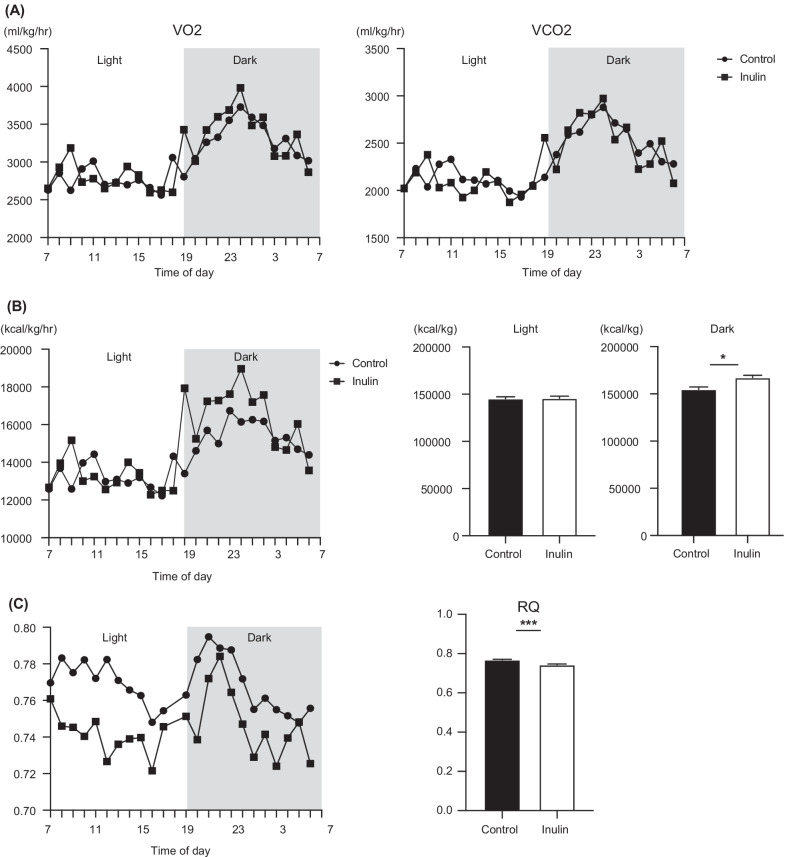
Effects of inulin on respiratory quotient, and energy expenditure in mice. **A** O_2_ consumption (VO_2_) and CO_2_ production (VCO_2_). **B** Energy expenditure during light and dark cycles. **C** Respiratory quotient, and energy expenditure. Light and dark cycles are indicated with white and gray backgrounds, respectively. Data are expressed as means ± SEM. *p < 0.05, ***p < 0.001. Control: n = 4; Inulin group: n = 4

The result of VO_2_ and VCO_2_ analyses are presented in Fig. 2A. We compared the energy expenditure in the inulin and control groups. Figure 2B indicates that the energy expenditure of the inulin group was significantly increased during the dark period (*p* = 0.013). In addition, after adjusting for body weight using ANCOVA, a significant difference was also observed during the dark period (*p* = 0.037).

The RQ (Fig. 2C) was found to be lower in the inulin group compared to the control group, thereby suggesting that in the inulin group, fat metabolism was higher than that of carbohydrate or protein during the dark and light periods.

### UCP1 expression of white adipose tissue

**Fig. 3:**
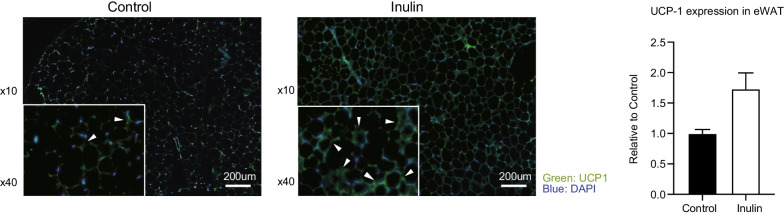
Immunostaining expression of UCP1 in epididymal white adipose tissue (eWAT). Scale bar = 200 µm. Data are expressed as means ± SEM. Control: n = 6; Inulin group: n = 6

The immunostaining analysis revealed that the expression of UCP1 in eWAT was elevated in the inulin group compared to the control group (Fig. 3). On the other hand, there was no difference in UCP1 expression between the two groups in sWAT (Additional file 2: Figure S1C).

### Inulin increases fecal and serum succinic acid and SCFA

**Fig. 4:**
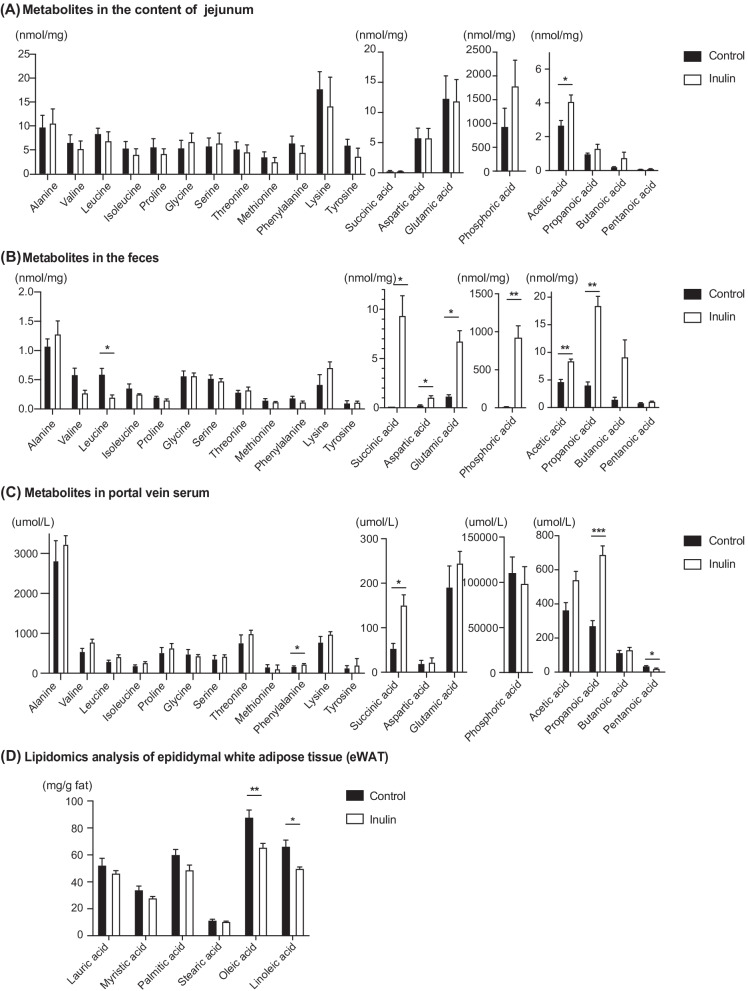
Results of the metabolomics analysis of the content of jejunum, feces, and portal vein serum. Lipidomics analysis. **A** Concentrations of amino acids, organic acids, and short-chain fatty acids (SCFAs) in the jejunum. **B** Concentrations of amino acids, organic acids, and short-chain fatty acids (SCFAs) in the feces. **C** Concentrations of amino acids, organic acids, and short-chain fatty acids (SCFAs) in the portal vein serum. **D** Lipidomics analysis of epididymal white adipose tissue (eWAT). Data are expressed as means ± SEM. *p < 0.05, **p < 0.01, ***p < 0.001. Control: n = 6; Inulin group: n = 6

We assessed the target metabolites in each sample using GC/MS. Among the metabolites in the jejunum, acetic acid of SCFA was higher in the inulin group (*p* = 0.077) compared to the control group (Fig. 4A). As a result of the metabolomics analysis of the rectal feces, succinic acid (*p* = 0.020), acetic acid (*p* = 0.008), and propanoic acid (*p* = 0.003) production increased significantly in the inulin group compared to the control group (Fig. 4B). Similarly, we found that the level of succinic acid (*p* = 0.033), acetic acid (*p* = 0.050), and propanoic acid (*p* < 0.001) in the portal vein serum were increased in the inulin group compared to the control group (Fig. 4C).

### Effect of inulin on fatty acid profile in visceral adipose tissue

Lipidomics analysis revealed that inulin decreased saturated fatty acids in eWAT. (Fig. 4D). The concentration of palmitic acid (*p* = 0.088), oleic acid (*p* = 0.009), and linoleic acid (*p* = 0.017) in eWAT were lower in the inulin group than those in the control.

## Discussion

Inulin and other soluble dietary fibers are highly fermentable and the role of gut metabolites, including SCFAs, has gained attention. Since the changes in the gut microbiota of Japanese diabetic patients were found to be significantly associated with the intake of a high-fat, and high-sucrose diet in our cohort study [21], we further aimed to investigate the effects of inulin supplementation in a high-fat, and high-sucrose diet fed mice model. No difference in the final body weight was observed between the two groups as a result of pair-feeding and equal caloric intake considering that inulin has an energy content of 1.5 kcal/g. However, there were significant differences in fat distribution in the body, gut metabolites, and metabolic activity between the two groups, which is suggestive.

As mentioned above, inulin produces SCFAs, succinic acid, and lactic acid via gut microbiota [3, 22, 23]. Consistent with previous reports [12], bacterial fermentation in the intestine increased the content of fecal SCFAs in the inulin group. There was a significant increase in the content of postprandial portal vein plasma succinic acid and SCFAs in the inulin group. The increase in amino acids, organic acids, and SCFAs in both the feces and serum in the inulin group reflected increased fermentation and absorption from the intestinal tract into the portal circulation. The reason why some metabolites were found to be increased in the feces but not in the serum could be that they were absorbed by the colon tissue [24, 25].

It is also noteworthy that succinic acid was found to be increased in the stool and portal vein serum of the inulin group. Consistent with previous reports, in the present study, inulin increased the level of succinic acid, a gut metabolite, in both the plasma and feces through bacterial fermentation. In regard with glucose metabolism, it is known that the increase in the content of SCFAs promotes the production of glucagon-like peptide-1 (GLP-1) from enteroendocrine cells and suppresses postprandial blood glucose elevation and appetite [26, 27], which may have resulted in a positive effect on the glucose metabolism in this study as well. In addition, it has been suggested that succinic acid and GLP-1 has synergistic insulinotropic effects [28].

Succinic acid is also shown to contribute to the improvement of glucose tolerance by acting as a substrate of intestinal glucogenesis and inhibits hepatic glucose production via intestinal glycogenesis [29]. Succinic acid from the microflora not only acts as a glucose precursor, but has been suggested to be akin to hormones as a reporter of metabolism and stress and an integrator of macrophage immune response [30].

In addition, succinate is known to activate heat production in adipose tissue and increase energy expenditure [8]. The underlying mechanism is that the oxidation of succinate promotes the production of mitochondrial reactive oxygen species (ROS) via succinate dehydrogenase oxidation [31, 32], which further leads to thermogenesis [8], thereby resulting in the acceleration of fat metabolism. It is suggested succinic acid produced in the intestine, after being absorbed in the body, further promotes fat metabolism and thermogenesis of visceral fat, which ultimately contributes to the reduction of visceral fat mass in the inulin group.

We observed a decrease in the visceral fat mass in the inulin group compared with the subcutaneous fat mass, and that the fatty acid profile of the visceral fat improved, thereby resulting in improved glucose tolerance in the individuals. As reported by Kimura et al. [33], SCFA exerts an inhibitory effect on fat accumulation via GPR43, a type of GPCR. Moreover, as a remarkable effect of SCFA on systemic metabolism, it has been confirmed that white adipose tissue (WAT) beiging and an increase in energy expenditure are positively induced upon SCFA supplementation [6]. These reports are in line with our results that the inulin group showed increased SCFA in the serum and the relative decrease in the visceral fat mass. Furthermore, the activation of UCP1 expression in visceral adipose tissue in the inulin group may have resulted in adipocyte beiging [34, 35], and an increase in energy expenditure in visceral fat.

Our results demonstrate two important points. First, inulin reduces visceral adipose tissue mass and improves glucose tolerance, even though it has no effect on whole body weight. Second, we evaluated the effect of inulin under the calorie-matched condition calculated by considering the calories produced by soluble fiber fermentation in the gut. This study provides new insight into the significant benefit of taking soluble fiber.

## Conclusions

Conclusively, our study revealed that inulin regulates energy metabolism and the adipocyte quality in visceral adipose tissue and improves glucose tolerance through altering gut metabolites.

## Supplementary Information

**Additional file 1: Table S1.** Composition of the control and inulin diets.

**Additional file 2: Figure S1.** **A** Weekly food intake. **B** Histological examination of subcutaneous white adipose tissues (sWAT) using hematoxylin and eosin (H&E) staining. **C** Immunostaining expression of UCP1 in subcutaneous white adipose tissue (sWAT). Scale bar = 200 µm. Data are expressed as means ± SEM. Control: n = 6; Inulin group: n = 6.

## Data Availability

The data that support the fndings of this study are available from the corresponding author upon reasonable request.

## Abbreviations

SCFAs: Short-chain fatty acids
GPCRs: G protein-coupled receptors
HFHSD: High-fat and high-sucrose diet
VO2: Oxygen consumption
VCO2: Carbon dioxide production
RQ: Respiratory quotients
IPGTT: Intraperitoneal glucose tolerance test
AUC: Area under the curve
eWAT: Epididymal white adipose tissue
sWAT: Subcutaneous white adipose tissue
DAPI: 4ʹ,6-Diamidino-2-phenylindole
GC/MS: Gas chromatography-mass spectrometry
SPE: Solid phase extraction
MSTFA: N-methyl-N-trimethylsilyltrifluoroacetamide
MTBSTFA: N-tert-butyldimethylsilyl-N-methyltrifluoroacetamide
ANCOVA: Analysis of covariance
GLP-1: Glucagon-like peptide-1
ROS: Reactive oxygen species
