# Supplementary material for: Inulin reduces visceral adipose tissue mass and improves glucose tolerance through altering gut metabolites
Source: Nutr Metab (Lond). 2022 Jul 28;19:50. doi: 10.1186/s12986-022-00685-1 (PMC9331483; doi:10.1186/s12986-022-00685-1)
Supplement: Supplementary file 1 — Additional file 1: Table S1. Composition of the control and inulin diets. [file 12986_2022_685_MOESM1_ESM.docx]

**Table S1.** Composition of diets

|  | **Control** | **Inulin** |
| --- | --- | --- |
| Protein (% kcal) | 20 | 20 |
| Carbohydrate (% kcal) | 40 | 40 |
| Fat (% kcal) | 40 | 40 |
| Energy (kcal/mg) | 4.60 | 4.02 |
| **Ingredient** |  |  |
| Corn starch (% kcal) | 0 | 0 |
| Maltodextrin 10 (% kcal) | 7.5 | 0 |
| Sucrose (% kcal) | 32 | 32 |
| Cellulose, BW 200 (% g) | 6 | 6 |
| Inulin (% kcal) | 0 | 7.5 |
| **(mg)** |  |  |
| Soybean Oil | 45 | 45 |
| Coconut Oil, 101 | 135 | 135 |
| Mineral Mix S10001 | 35 | 35 |
| Calcium Carbonate | 5 | 5 |
| Vitamin Mix V10001 | 10 | 10 |
| Choline Bitartrate | 2 | 2 |
