# Supplementary figures and images for: Inulin reduces visceral adipose tissue mass and improves glucose tolerance through altering gut metabolites
Source: Nutr Metab (Lond). 2022 Jul 28;19:50. doi: 10.1186/s12986-022-00685-1 (PMC9331483; doi:10.1186/s12986-022-00685-1)

FigureS1

(A)

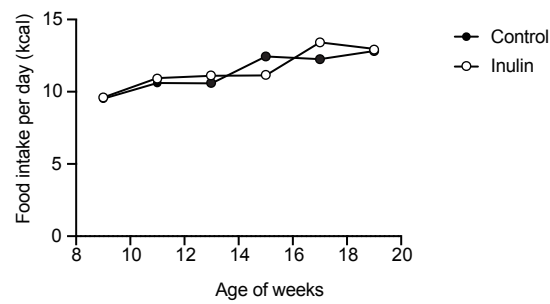

(B)

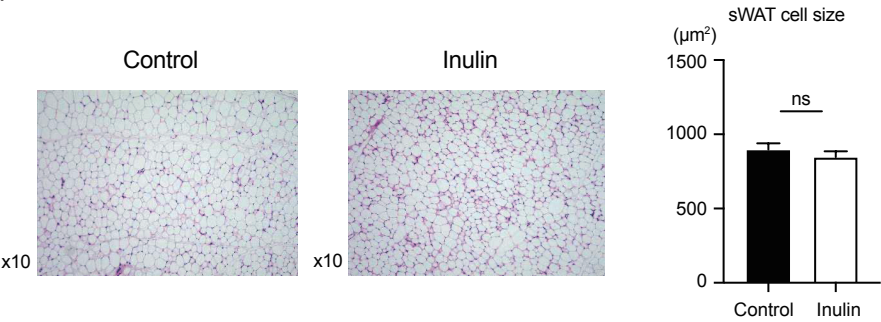

(C)

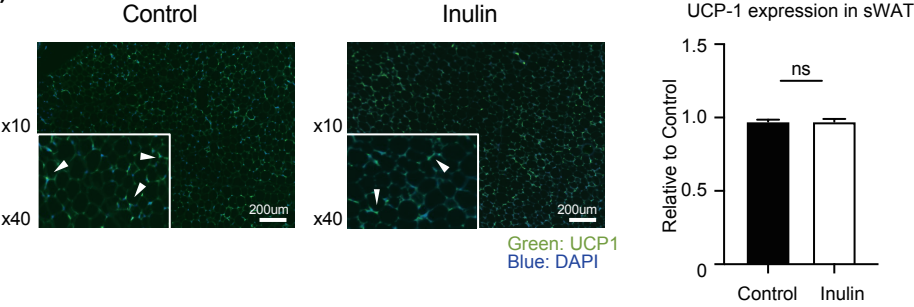

Supplement: Supplementary file 2 — Additional file 2: Figure S1. A Weekly food intake. B Histological examination of subcutaneous white adipose tissues (sWAT) using hematoxylin and eosin (H&E) staining. C Immunostaining expression of UCP1 in subcutaneous white adipose tissue (sWAT). Scale bar = 200 µm. Data are expressed as means ± SEM. Control: n = 6; Inulin group: n = 6. [file 12986_2022_685_MOESM2_ESM.pdf]
